# Empowering leadership and university teachers’ work engagement: the mediating role of job crafting and the moderating role of role breadth self-efficacy

**DOI:** 10.3389/fpsyg.2026.1929988

**Published:** 2026-09-18

**Authors:** Dian-Min Xiao, Guang-Hui Wang, Zi-Yi Huang, Duo-Qin Huang

**Affiliations:** 1 The First Affiliated Hospital of Gannan Medical University, Ganzhou, Jiangxi, China; 2 School of Educational Science, Guangdong Polytechnic Normal University, Guangzhou, Guangdong, China; 3 School of Public Health and Health Management, Gannan Medical University, Ganzhou, Jiangxi, China; 4 The First Clinical Medical College of Gannan Medical University, Ganzhou, Jiangxi, China

**Keywords:** empowering leadership, job crafting, job demands–resources theory, role breadth self-efficacy, work engagement

## Abstract

**Introduction:**

University teachers work under growing workload, performance, and role pressures, making work engagement an important concern for higher education institutions. Drawing on job demands–resources theory, this study examined whether job crafting mediates the association between empowering leadership and work engagement and whether role breadth self-efficacy (RBSE) moderates this indirect relationship among university teachers.

**Methods:**

Using a cross-sectional survey design, data were collected from 468 university teachers in China. The hypothesized mediation and moderated mediation relationships were examined using path analysis.

**Results:**

Empowering leadership was positively associated with work engagement, and job crafting mediated this relationship. RBSE strengthened the association between empowering leadership and job crafting, such that the indirect association between empowering leadership and work engagement through job crafting was stronger among teachers with higher RBSE.

**Discussion:**

The findings are consistent with an indirect relationship between empowering leadership and work engagement through proactive changes in tasks, relationships, and work meaning. The study extends job demands–resources theory to the higher education context and highlights empowering leadership, support for job crafting, and the development of RBSE as potential practical approaches for sustaining university teachers’ work engagement.

## 1 Introduction

University teachers are expected to be disciplinary experts and to invest sustained energy in teaching, research, student support, service, and public engagement. Work engagement is commonly defined as a positive and fulfilling work-related state marked by vigor, dedication, and absorption (Schaufeli et al., 2006). In universities, engagement matters because academic work is knowledge-intensive and partly discretionary. Teachers decide how much initiative to invest in course design, student mentoring, collaborative research, grant applications, and institutional service (Whitsed et al., 2025). At the same time, academic staff face heavier workloads, publication pressure, complex student-support demands, work–life conflict, and performance-management systems, while autonomy, social support, meaningful work, and leadership support remain central resources for well-being (Cao and Zhang, 2022; Mudrak et al., 2018; Naidoo-Chetty and du Plessis, 2021; Sabagh et al., 2018). These conditions make university teaching a useful context for examining how leadership practices foster sustained engagement (Huang and Wang, 2026; Rinas et al., 2022).

The job demands-resources (JD-R) perspective offers the theoretical starting point. JD-R theory proposes that work engagement is stimulated when job resources help employees attain work goals and reduce the psychological costs of job demands (Bakker and Demerouti, 2017). Meta-analytic and longitudinal evidence supports this motivational pathway from resources to engagement and also shows that job demands have different effects depending on whether they function as challenges or hindrances (Crawford et al., 2010; Lesener et al., 2019; Mazzetti et al., 2023). Recent research further treats demands and resources as part of dynamic and proactive processes, not merely as static background conditions (Kwon and Kim, 2020). This point is especially relevant in universities, where roles are structured by departments and institutional policies but enacted through considerable professional discretion. Academic leaders may therefore influence engagement not only by supplying resources directly, but also by shaping whether teachers feel trusted, capable, and authorized to use those resources proactively. Because empowering leadership is designed to share power, encourage participation, and express confidence in employees, it is closely aligned with this resource-based process (Lee et al., 2018).

Empowering leadership is particularly relevant in universities because it joins discretion with trust in professional competence. Empowering leaders enhance work meaningfulness, encourage participation in decision making, express confidence in followers’ performance, and provide autonomy from unnecessary bureaucratic constraints (Ahearne et al., 2005). Meta-analytic and review evidence links empowering leadership to employee motivation, attitudes, and performance, although its effects depend on the mechanisms and boundary conditions through which empowerment is experienced (Cheong et al., 2019; Lee et al., 2018). Li et al. (2021) similarly show that leadership is reliably associated with engagement, while the strength of this association varies across leadership forms and national-cultural contexts. In universities, empowering leadership may be enacted by deans, department heads, program directors, or research-team leaders who consult teachers on decisions, communicate confidence in their professional judgment, and give them latitude to design meaningful approaches to teaching, research, and service. Such behavior is important because academic work often combines formal professional autonomy with practical administrative constraint.

The link between empowering leadership and engagement is unlikely to be only direct. A key question is how leadership resources such as discretion, confidence, and autonomy become teachers’ proactive work redesign. Job crafting provides a behavioral explanation. It refers to self-initiated changes through which employees alter the task, relational, or cognitive boundaries of work so that jobs better fit their strengths, motives, and values (Wrzesniewski and Dutton, 2001). From a JD-R perspective, job crafting also describes employees’ proactive modification of job resources and demands, including increasing structural and social resources, seeking challenging demands, and reducing hindering demands (Tims et al., 2022; Zhang and Parker, 2019). Meta-analytic evidence links job crafting, especially resource- and challenge-oriented crafting, to work engagement (Rudolph et al., 2017). For university teachers, crafting may involve redesigning teaching methods, experimenting with assessment practices, seeking interdisciplinary collaboration, reframing routine service as student development, or building developmental relationships with colleagues. These examples fit integrative accounts that distinguish task, relational, skill, and cognitive changes and separate approach-oriented from avoidance-oriented crafting (Bindl et al., 2019; Bruning and Campion, 2018; Lazazzara et al., 2020). Job crafting therefore explains how empowering leadership may be translated into engagement through teachers’ active shaping of their work.

A further boundary condition is whether teachers have the personal resources needed to use empowerment proactively. Within the JD-R framework, empowering leadership can be treated as a job resource because it provides autonomy, participative influence, confidence from academic leaders, and reduced bureaucratic constraints (Bakker and Demerouti, 2024). Yet these resources do not automatically produce job crafting. Their motivational value may depend on personal resources, which shape whether discretion is experienced as an opportunity for work redesign or as an additional demand. JD-R research explicitly includes personal resources, including self-efficacy, as factors that help employees influence their work environment and mobilize available job resources (Xanthopoulou et al., 2007). Role breadth self-efficacy (RBSE), defined as confidence in carrying out broader and more proactive work activities beyond narrow role requirements, is therefore a theoretically relevant personal resource (Parker, 1998). In academic work, teachers with higher RBSE should be better prepared to translate empowering leadership into job crafting because they feel capable of taking initiative across teaching, research, student support, collegial collaboration, and institutional service. Teachers with lower RBSE may instead experience expanded discretion as ambiguity or responsibility without sufficient confidence to act.

Prior research has linked empowering leadership to employee well-being through job crafting, with leadership and core self-evaluations examined as parallel antecedents (Kim and Beehr, 2020). Thun and Bakker (2018) further showed that optimism strengthened the associations of empowering leadership with increasing structural resources and challenging demands. More recently, Mao et al. (2026) positioned RBSE as a mediator between empowering leadership and job crafting. These studies establish the relevance of personal resources but leave their role in followers’ responses to empowerment incompletely specified. In particular, explaining efficacy as a mechanism linking leadership to crafting differs from explaining whether efficacy conditions the strength of that relationship. Likewise, generalized expectations of favorable outcomes are distinct from confidence in undertaking the broader activities involved in work redesign. What remains insufficiently understood is whether role-specific efficacy complements empowering leadership within the job crafting pathway to engagement. This question matters because leadership may provide opportunities for initiative without those opportunities being equally actionable for all followers. Examining RBSE as a boundary condition therefore addresses whether the indirect association between empowering leadership and work engagement depends on followers’ perceived capability to act beyond narrowly defined roles. This issue is particularly relevant to university teachers, for whom professional discretion and confidence in assuming broader responsibilities need not coincide.

The present study tests this moderated mediation model in a sample of university teachers. We propose that empowering leadership is positively related to work engagement, that job crafting mediates this relationship, and that RBSE strengthens the first-stage relationship between empowering leadership and job crafting. The study offers several contributions. First, it extends research on work engagement in higher education by conceptualizing empowering leadership as a job resource that is associated with engagement partly through teachers’ proactive agency, rather than as a simple top-down predictor. Second, it connects empowering leadership and job crafting research by examining whether leader-provided discretion, confidence, and participation are associated with bottom-up work redesign. Third, it clarifies RBSE as a personal resource that moderates the relationship between empowering leadership and job crafting. Together, these contributions advance JD-R-based explanations of academic work by examining how job resources, personal resources, and job crafting are jointly associated with university teachers’ engagement in demanding institutional environments (Bakker and Demerouti, 2024; Granziera et al., 2022; Naidoo-Chetty and du Plessis, 2021).

The university context also sharpens the theoretical contribution by challenging a common assumption in empowerment research. Empowerment is often framed as a shift from leader control to employee discretion. In universities, professional autonomy and institutional control often coexist (Puaca, 2021). Teachers may have discretion over pedagogical methods, research questions, and professional collaboration while facing publication metrics, teaching evaluations, accreditation requirements, and administrative reporting demands (Mudrak et al., 2018; Naidoo-Chetty and du Plessis, 2021; Sabagh et al., 2018). In this setting, empowerment is not merely formal delegation of authority. It is a local relational process through which academic leaders help teachers interpret discretion, prioritize meaningful goals, and feel able to take initiative. By locating the model in university teachers’ work, this study examines empowerment in a setting where autonomy is already part of professional identity but still requires leadership support to become proactive behavior and sustained engagement.

## 2 Theory and hypotheses

### 2.1 Empowering leadership and work engagement

Empowering leadership refers to leader behaviors that increase followers’ opportunity, ability, and motivation to influence their work. Ahearne et al. (2005) identify four key behaviors: enhancing work meaningfulness, encouraging participation in decision making, expressing confidence in high performance, and providing autonomy from bureaucratic constraints. Engagement-leadership research also suggests that leaders can foster engagement by facilitating autonomy, strengthening competence, connecting employees, and creating meaning (Schaufeli, 2021). In JD-R terms, these behaviors are not simply supportive interpersonal acts. They are job resources that provide voice, discretion, competence-related feedback, and meaningful goal cues. In universities, such leadership may help teachers experience their academic roles as self-directed professional contributions rather than as compliance with administrative requirements.

JD-R theory provides the main rationale for expecting empowering leadership to enhance engagement. Job resources stimulate engagement because they help employees achieve work goals, manage demands, and support growth and development (Bakker and Demerouti, 2017; Mazzetti et al., 2023). Empowering leadership should matter for three reasons. First, autonomy and participation are central job resources. When teachers are consulted about curricular, research, or departmental decisions, they are more likely to feel that their professional judgment matters and that they can influence decisions affecting their core work. Second, empowering leadership expresses confidence in teachers’ capabilities. This confidence functions as both an informational and a socio-emotional resource, increasing energy and persistence during complex teaching or research tasks. Third, empowering leadership enhances work meaningfulness by linking teachers’ goals to broader departmental and university missions. Meaningfulness is closely aligned with dedication, the engagement dimension involving enthusiasm, pride, and a sense of significance, and teacher research shows that meaning at work can strengthen engagement while reducing strain (Lavy, 2022; Schaufeli et al., 2006).

Evidence from JD-R research and higher education supports this reasoning. Leadership can function as a social and organizational job resource that stimulates the motivational process leading to engagement (Bakker and Demerouti, 2017; Mazzetti et al., 2023). A JD-R-based multilevel study also shows that empowering leadership can optimize work conditions for engagement by shaping the resources and demands employees experience (Tuckey et al., 2012). In higher education, academic staff engagement depends on resources at individual, team, and organizational levels, including supportive leadership, collegial relations, recognition, and workable institutional processes. By contrast, workload pressure, inadequate resources, and managerial change are repeatedly reported as threats to well-being and motivation (Bakker and Demerouti, 2017; Whitsed et al., 2025). Because empowering leadership increases teachers’ access to voice, discretion, and confidence, it should strengthen the resource base from which engagement develops. This expectation is consistent with meta-analytic evidence that empowering leadership predicts favorable employee attitudes and motivation across organizational contexts (Cheong et al., 2019; Lee et al., 2018), as well as cross-national evidence that leadership is reliably associated with employee engagement (Li et al., 2021).

This argument does not assume that empowerment is beneficial in every circumstance. Reviews of empowering leadership note that its effects may vary when empowerment creates ambiguity or when followers lack the ability or motivation to use discretion effectively (Cheong et al., 2019). Academic work, however, typically requires specialized expertise, judgment, adaptation, and initiative. Empowering leadership should therefore fit the nature of academic work. When university teachers perceive that leaders trust their judgment, reduce unnecessary constraints, and invite meaningful participation, they are more likely to show vigor, dedication, and absorption. Accordingly, we propose:

*H1*: Empowering leadership will be positively related to work engagement.

### 2.2 The mediating role of job crafting

Although empowering leadership may directly foster work engagement, its influence should also operate through job crafting. Within the JD-R framework, job crafting is a bottom-up process through which employees proactively modify job resources and demands to improve person–job fit and work motivation. The concept also describes how employees shape tasks, relationships, and cognitions to make work more meaningful and better aligned with personal strengths and values (Wrzesniewski and Dutton, 2001). JD-R-based job crafting research further emphasizes proactive efforts to increase structural resources, increase social resources, seek challenging demands, and reduce hindering demands (Tims et al., 2012). Across these traditions, job crafting highlights employee agency in work design. Rather than treating university teachers as passive recipients of institutional arrangements, this perspective recognizes that teachers can modify how they teach, collaborate, seek feedback and support, manage administrative demands, and connect academic tasks to broader purposes.

Empowering leadership should stimulate job crafting because it gives teachers the contextual resources and motivational cues needed to redesign work. Leaders who invite participation signal that teachers’ opinions matter, which may encourage them to identify local problems and craft new solutions. Leaders who express confidence in teachers’ performance may increase willingness to experiment with new teaching practices or research collaborations. Leaders who simplify rules and provide autonomy reduce the perceived risk of departing from rigid routines. Empowering leadership therefore creates a work context in which crafting is legitimate, feasible, and valued. Kim and Beehr (2020) found that empowering leadership was positively related to expansive forms of job crafting, which subsequently predicted employee well-being outcomes. Daily research also shows that leader empowering behavior is associated with employees’ day-level job crafting, supporting leadership as a proximal trigger of proactive work redesign (Jiang et al., 2021). These findings fit reviews suggesting that empowering leadership often works through motivational and proactive mechanisms rather than through simple compliance pathways (Cheong et al., 2019; Lee et al., 2018).

Job crafting, in turn, should foster engagement because it enables teachers to build resources, pursue constructive challenges, and strengthen the fit between academic work and professional identity. Through task crafting, teachers can align teaching methods, assessment practices, supervision, or research projects with their expertise and interests. Through relational crafting, they can build support networks with colleagues, students, industry partners, or interdisciplinary collaborators. Through cognitive crafting, they can reinterpret routine teaching, advising, or committee work as connected to student development, scholarly identity, or institutional contribution. These forms of crafting can increase the resources, challenge, and meaning that energize engagement. Meta-analytic evidence supports positive associations between job crafting and engagement across occupations, especially for approach- or promotion-oriented crafting (Lichtenthaler and Fischbach, 2019; Rudolph et al., 2017). Recent meta-analytic evidence further indicates that job crafting affects employee outcomes partly by changing job characteristics, particularly task and social resources (Holman et al., 2024).

Recent developments further refine the expected mediation. Zhang and Parker (2019) distinguish approach-oriented crafting, which expands resources and meaning, from avoidance-oriented crafting, which may not have the same benefits. Bruning and Campion (2018) similarly show that role- and resource-based forms of crafting differ in their links with engagement and strain, while Tims et al. (2022) call for closer attention to motives, context, social relationships, and longitudinal processes. This distinction matters for university teachers because crafting stimulated by empowering leadership is likely to be primarily approach-oriented. Empowering leaders do not merely encourage teachers to withdraw from unpleasant tasks. They provide autonomy, confidence, and meaningful participation that should encourage teachers to seek resources, take on constructive challenges, and reframe work more positively. Research on active job changes also suggests that crafting is best understood as a differentiated set of task, relational, skill, and cognitive strategies rather than as a single generic behavior (Bindl et al., 2019; Lazazzara et al., 2020).

The mediating role of job crafting is especially plausible in universities. Academic jobs often contain high discretion and ambiguous boundaries, as teachers’ work spans teaching, research, service, student support, and administration within increasingly demanding workload and performance-accountability systems (Houston et al., 2006; Naidoo-Chetty and du Plessis, 2021). Teachers can vary in how they design classroom interaction, integrate research into teaching, build peer relationships, mentor students, participate in committees, or reinterpret service as professional contribution. Higher education research shows that university professionals use task, relational, and cognitive crafting to create meaning within complex institutional roles, and that approach-oriented crafting among university professors is linked to engagement and prosocial service behavior (Castiello-Gutiérrez et al., 2021; Ortega-Egea et al., 2025). Empowering leadership may provide the relational and organizational conditions for teachers to use this discretion productively, while job crafting explains the behavioral process through which those conditions become engagement. From a JD-R perspective, empowering leadership can be understood as a contextual job resource, job crafting as bottom-up resource building, and work engagement as the motivational outcome of more usable resources, challenges, and meaning. Taken together, we propose the following hypothesis.

*H2*: Job crafting mediates the relationship between empowering leadership and work engagement.

### 2.3 The moderating effect of role breadth self-efficacy

Empowering leadership may not lead all teachers to craft their jobs to the same extent. Although it provides discretion, voice, and confidence, these resources must be interpreted as usable and enacted in daily work. Within the JD-R framework, personal resources shape how employees perceive and mobilize job resources. RBSE captures individuals’ confidence that they can carry out broader and more proactive work activities beyond narrowly specified role requirements (Parker, 1998). It differs from general self-efficacy because it focuses on confidence in assuming wider role responsibilities and acting beyond prescribed tasks. In academic work, RBSE may include confidence in analyzing long-term problems, influencing departmental processes, initiating new teaching or research practices, and contributing across teaching, research, student support, collegial collaboration, and institutional service. Prior research links RBSE to proactive behavior directed toward teams and organizations, and more recent work connects it with innovative work behavior and broader career-relevant outcomes (Rodrigues and Rebelo, 2023; Strauss et al., 2009).

The moderating logic follows the JD-R distinction between job resources and personal resources. JD-R research treats personal resources, including self-efficacy, as factors that influence how employees perceive their work environment, use available job resources, and sustain motivation (Xanthopoulou et al., 2007). In this study, empowering leadership represents a contextual job resource, whereas RBSE represents a personal resource that should strengthen teachers’ ability to convert leader-provided discretion into job crafting. Teachers with high RBSE should be more likely to interpret empowering leadership as an opportunity to expand resources and take constructive initiative. They should feel better able to redesign tasks, seek developmental relationships, experiment with new pedagogical or research practices, and reframe academic responsibilities in more meaningful ways. Teachers with lower RBSE may appreciate supportive leadership but still avoid proactive crafting when empowerment increases ambiguity, responsibility, or the perceived risk of acting beyond established routines. This reasoning is consistent with evidence that RBSE conditions the effects of empowering leadership on employee outcomes and with work showing that empowering leadership can be enabling or burdening depending on how followers use discretion (Cheong et al., 2016; Hao et al., 2018).

The academic context makes this interaction particularly salient. University teachers often occupy broad, boundary-spanning roles that include teaching, research, student support, collegial collaboration, institutional service, and responses to performance-related demands (Mudrak et al., 2018; Naidoo-Chetty and du Plessis, 2021). Such roles create opportunities for job crafting, but they also require teachers to decide where to invest effort and how far to extend their roles. Teachers with high RBSE should be more capable of using empowering leadership to cross role boundaries productively, for example by proposing curricular changes, building interdisciplinary collaborations, redesigning mentoring routines, or reframing service as professional contribution. Teachers with low RBSE may perceive the same empowerment as uncertain or demanding, especially when workloads are heavy or performance expectations are ambiguous. Thus, RBSE should amplify the positive effect of empowering leadership on job crafting by increasing the likelihood that leader-provided discretion becomes resource-generating action. Accordingly, we propose:

*H3*: The positive relationship between empowering leadership and job crafting is moderated by role breadth self-efficacy, such that the relationship is stronger when role breadth self-efficacy is higher.

Extending the mediation and moderation arguments above, we expect RBSE to condition the indirect relationship between empowering leadership and work engagement through job crafting. This pattern represents a first-stage moderated mediation model because RBSE is expected to moderate the path from empowering leadership to job crafting, thereby changing the size of the indirect effect on work engagement (Preacher et al., 2007). When RBSE is high, university teachers should be more likely to interpret empowering leadership as a usable resource and convert leader-provided autonomy, voice, and confidence into efforts to build resources, seek constructive challenges, and shape work more meaningfully. These job crafting behaviors should then increase the resources, challenge, and meaning that foster engagement. When RBSE is low, empowering leadership may still be experienced as supportive, but teachers may be less likely to transform that support into active work redesign. The indirect effect of empowering leadership on work engagement through job crafting should therefore be weaker.

This conditional indirect effect is consistent with JD-R theory. In the present model, empowering leadership represents a contextual job resource, RBSE represents a personal resource, job crafting represents bottom-up resource building, and work engagement represents the motivational outcome of the resource process (Bakker and Demerouti, 2024; Xanthopoulou et al., 2007). RBSE matters because the mediator is not an automatic psychological state but an agentic behavior. Teachers are more likely to transform leader-enabled discretion into job crafting when they feel capable of acting beyond narrow role boundaries. Thus, empowering leadership should lead to stronger engagement through job crafting when teachers have high rather than low RBSE. Accordingly, we propose:

*H4*: Role breadth self-efficacy moderates the indirect relationship between empowering leadership and work engagement via job crafting, such that the indirect relationship is stronger for teachers with high rather than low role breadth self-efficacy.

Taken together, the framework proposes a conditional resource pathway in which empowering leadership functions as a contextual job resource associated with work engagement both directly and indirectly through job crafting, while RBSE operates as a personal resource that strengthens the empowering leadership–job crafting relationship and, consequently, the indirect association with work engagement (see Figure 1).

**Figure 1:**
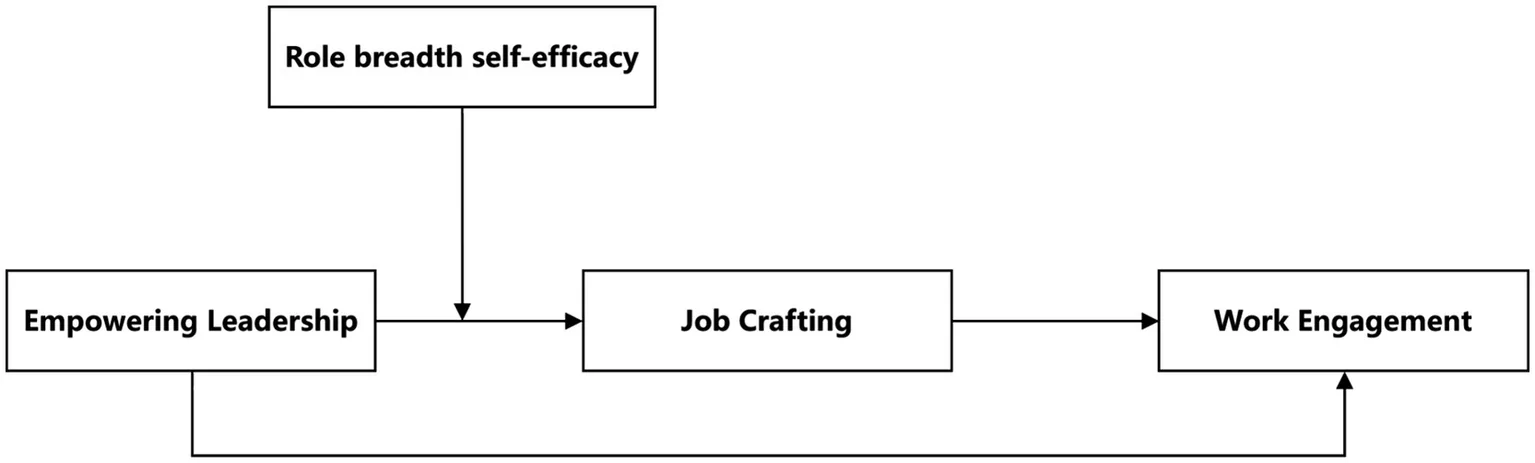
Empowering leadership is positively associated with work engagement directly and indirectly through job crafting. RBSE moderates the relationship between empowering leadership and job crafting, such that the indirect association with work engagement is stronger at higher levels of RBSE.

## 3 Methods

### 3.1 Sample and procedure

Participants were recruited through convenience sampling from the target population of university faculty members in Chinese higher education institutions. Convenience sampling was adopted because it facilitated access to eligible university teachers. The questionnaire was administered online through Wenjuanxing, and the survey link was distributed to university teachers across multiple higher education institutions. Data were collected through a voluntary questionnaire survey. Before participation, respondents were informed about the study purpose, data collection procedures, intended use of the data, and the anonymous and confidential handling of their responses. Participation was entirely voluntary, and informed consent was obtained before respondents completed the survey.

The final sample included 468 valid responses. Of the respondents, 228 were male (48.7%) and 240 were female (51.3%). The mean age was 43.68 years (SD = 7.90). In terms of educational background, 6 participants held a bachelor’s degree (1.3%), 97 held a master’s degree (20.7%), and 365 held a doctoral degree (78.0%). Regarding academic rank, 43 respondents were teaching assistants (9.2%), 182 were lecturers (38.9%), 160 were associate professors (34.2%), and 83 were professors (17.7%). Organizational tenure was defined as length of employment at the current institution: 120 respondents had worked for fewer than 5 years (25.6%), 168 for 5 to 10 years (35.9%), and 180 for more than 10 years (38.5%).

With respect to institutional type, 173 participants worked at universities included in China’s national “Double First-Class University” construction scheme (37.0%), whereas 295 worked at other regular higher education institutions (63.0%). The sample covered several academic disciplines: Humanities and Social Sciences (*n* = 79, 16.9%), Medical Sciences (*n* = 245, 52.4%), Engineering and Technology Sciences (*n* = 66, 14.1%), Agricultural Sciences (*n* = 24, 5.1%), and Natural Sciences (*n* = 54, 11.5%). Although the sample included teachers from different institutional types, academic ranks, and disciplines, it should not be considered nationally representative of Chinese university teachers. The study was reviewed and approved by the Ethics Review Committee of The First Affiliated Hospital of Gannan Medical University, with ethics approval decision number LLSC-2026283.

### 3.2 Measures

#### 3.2.1 Empowering leadership

We measured empowering leadership using a 12-item scale adapted from Ahearne et al. (2005). All items were measured on a five-point Likert scale (1 = *strongly disagree* to 5 = *strongly agree*). The scale assesses four dimensions: (a) enhancing the meaningfulness of work (three items, *α* = 0.834; example item: “My leader helps me understand how my job fits into the bigger picture”), (b) fostering participation in decision making (three items, *α* = 0.871; example item: “My leader often consults me on strategic decisions”), (c) expressing confidence in high performance (three items, *α* = 0.884; example item: “My leader expresses confidence in my ability to perform at a high level”), and (d) providing autonomy from bureaucratic constraints (three items, *α* = 0.893; example item: “My leader allows me to do my job my way”). The Cronbach’s alpha in the current study was 0.953.

#### 3.2.2 Job crafting

We measured job crafting using the 15-item scale developed by Slemp and Vella-Brodrick (2013). The scale assesses three dimensions: (a) task crafting (five items, *α* = 0.930; example item: “Introduce new approaches to improve your work”), (b) cognitive crafting (five items, *α* = 0.922; example item: “Think about how your job gives your life purpose”), and (c) relational crafting (five items, *α* = 0.933; example item: “Organise or attend work related social functions”). Responses ranged from 1 = hardly ever to 5 = very often. The Cronbach’s alpha in the current study was 0.972.

#### 3.2.3 Role breadth self-efficacy

Role breadth self-efficacy was measured using a 10-item scale developed by Parker (1998). Respondents indicated how confident they would feel in carrying out broader and more proactive work activities in their university work. A sample item is “Analyzing a long-term problem to find a solution.” Responses were rated on a five-point scale ranging from 1 = not at all confident to 5 = very confident. The Cronbach’s alpha in the current study was 0.937.

#### 3.2.4 Work engagement

Work engagement was assessed using the 9-item short version of the Utrecht Work Engagement Scale (Schaufeli et al., 2006). The scale consists of three sub-dimensions (three items each): Vigor (e.g., “At my job, I feel strong and vigorous”), Dedication (e.g., “My job inspires me”), and Absorption (e.g., “I feel happy when I am working intensely”). Responses were measured on a 5-point frequency scale (1 = Never to 5 = Always). In this study, the Cronbach’s alpha coefficients for the sub-dimensions were 0.776 for Vigor, 0.851 for Dedication, and 0.831 for Absorption. The Cronbach’s alpha for the overall scale in the present study was 0.927.

#### 3.2.5 Control variables

Because demographic characteristics may influence teachers’ work engagement, gender, age, and organizational tenure were included as control variables.

### 3.3 Data analysis

Descriptive statistics, Cronbach’s alpha coefficients, and Pearson correlations were calculated using SPSS 26.0. Confirmatory factor analyses were conducted using Mplus 8.3 to assess the distinctiveness of the four focal constructs. A common method factor model was also estimated in Mplus 8.3 to assess potential common method variance. Regression-based path analysis was conducted using the PROCESS macro for SPSS to examine the direct association between empowering leadership and work engagement, the mediating role of job crafting, and the moderating role of RBSE in the empowering leadership–job crafting relationship. Conditional indirect effects were estimated at RBSE values one standard deviation above and below the mean. Moderated mediation was evaluated using the index of moderated mediation and its 95% confidence interval.

## 4 Results

### 4.1 Descriptive statistics and correlations

Table 1 reports the means, standard deviations, and zero-order correlations among the variables. The four focal variables had relatively high mean scores: empowering leadership (M = 3.948, SD = 0.751), job crafting (M = 3.865, SD = 0.758), role breadth self-efficacy (M = 3.792, SD = 0.792), and work engagement (M = 3.696, SD = 0.760). Empowering leadership was positively correlated with work engagement (*r* = 0.376, *p* < 0.001) and job crafting (*r* = 0.309, *p* < 0.001). Job crafting was also positively correlated with work engagement (*r* = 0.651, *p* < 0.001). Role breadth self-efficacy was positively correlated with job crafting (*r* = 0.488, *p* < 0.001) and work engagement (*r* = 0.337, *p* < 0.001), and showed a small positive correlation with empowering leadership (*r* = 0.096, *p* < 0.05). Overall, the bivariate relationships among the main variables were consistent with the proposed theoretical model.

**Table 1:** Descriptive statistics and correlations.

Variables	1	2	3	4	5	6	7
1. Gender	-						
2. Age	−0.055	-					
3. Organizational tenure	−0.020	0.795^***^	-				
4. Empowering leadership	−0.001	0.087	0.080	-			
5. Job crafting	0.068	0.033	0.055	0.309^***^	-		
6. Role breadth self-efficacy	−0.009	0.014	0.010	0.096^*^	0.488^***^	-	
7. Work engagement	0.051	0.001	0.010	0.376^***^	0.651^***^	0.337^***^	-
M	1.513	43.677	2.128	3.948	3.865	3.792	3.696
SD	0.500	7.897	0.791	0.751	0.758	0.792	0.760

*N* = 468. ^*^ *p* < 0.05, ^**^ *p* < 0.01, ^***^ *p* < 0.001. Gender was coded as 1 = male, 2 = female. Age was recorded as the actual age in years. Organizational tenure was coded as 1 = less than 5 years, 2 = 5 to 10 years, 3 = more than 10 years.

### 4.2 Confirmatory factor analysis

Before testing the hypotheses, confirmatory factor analysis was conducted to examine the discriminant validity of the four focal constructs: empowering leadership, job crafting, role breadth self-efficacy, and work engagement. The hypothesized four-factor model, in which the four constructs were specified as distinct latent variables, fit the data well: *χ*^2^ = 155.100, df = 59, *χ*^2^/df = 2.629, CFI = 0.984, TLI = 0.979, RMSEA = 0.059, and SRMR = 0.022. This model fit substantially better than the alternative three-factor model combining empowering leadership and job crafting, *χ*^2^ = 1540.616, df = 62, *χ*^2^/df = 24.849, CFI = 0.753, TLI = 0.689, RMSEA = 0.226, and SRMR = 0.200. It also outperformed the two-factor model combining empowering leadership with job crafting and role breadth self-efficacy with work engagement, *χ*^2^ = 2175.515, df = 64, *χ*^2^/df = 33.992, CFI = 0.647, TLI = 0.570, RMSEA = 0.266, and SRMR = 0.215, as well as the one-factor model combining all focal constructs, *χ*^2^ = 3245.964, df = 65, *χ*^2^/df = 49.938, CFI = 0.468, TLI = 0.362, RMSEA = 0.323, and SRMR = 0.205. These results indicate that the hypothesized four-factor measurement model best represented the data and that the four constructs were empirically distinguishable. To assess potential common method variance, we additionally estimated a model incorporating a common method factor (CMF) alongside the four substantive factors (Podsakoff et al., 2012). As shown in Table 2, the CMF model demonstrated good fit: *χ*^2^(47) = 125.291, CFI = 0.987, TLI = 0.978, RMSEA = 0.060, and SRMR = 0.015. Compared with the original four-factor model, CFI increased by 0.003 and SRMR decreased by 0.007, whereas TLI decreased by 0.001 and RMSEA increased by 0.001. Although these changes in fit indices were modest, they do not establish the absence of common method variance or its impact on the estimated relationships. Accordingly, potential common method bias remains a limitation when interpreting the findings.

**Table 2:** Confirmatory factor analysis.

Model	Factors	*χ* ^2^	df	*χ*^2^/df	CFI	TLI	RMSEA	SRMR
Four-factor model	EL, JC, RBSE, WE	155.100	59	2.629	0.984	0.979	0.059	0.022
Three-factor model	EL + JC, RBSE, WE	1540.616	62	24.849	0.753	0.689	0.226	0.200
Two-factor model	EL + JC, RBSE+WE	2175.515	64	33.992	0.647	0.570	0.266	0.215
One-factor model	EL + JC + RBSE+WE	3245.964	65	49.938	0.468	0.362	0.323	0.205
Five-factor model	EL, JC, RBSE, WE, CMF	125.291	47	2.666	0.987	0.978	0.060	0.015

EL, empowering leadership. JC, job crafting. RBSE, role breadth self-efficacy. WE, work engagement. CMF, common method factor.

### 4.3 Hypothesis testing

Before testing the hypotheses, multicollinearity among the focal predictors was assessed using variance inflation factors (VIFs). The VIFs for empowering leadership, job crafting, RBSE, and the interaction term were 1.46, 1.47, 1.36, and 1.35, respectively, indicating that multicollinearity among the focal predictors was not a concern.

Hypothesis 1 predicted a positive association between empowering leadership and work engagement. As shown in Table 3, empowering leadership was positively related to work engagement (B = 0.199, *p* < 0.001, *β* = 0.197), supporting Hypothesis 1.

**Table 3:** Results of path analysis.

Variables	*B*	*β*	95% CI
Direct path			
EL → WE	0.199	0.197	[0.127, 0.271]
Indirect path			
EL → JC → WE	0.184	0.182	[0.116, 0.263]
EL → JC → WE			
+1 SD RBSE	0.367	0.363	[0.275, 0.461]
−1 SD RBSE	0.043	0.043	[−0.015, 0.121]
Index	0.205	0.160	[0.134, 0.262]

B, unstandardized estimate; *β*, standardized estimate; CI, confidence interval. Confidence intervals refer to unstandardized estimates. EL, empowering leadership. JC, job crafting. RBSE, role breadth self-efficacy. WE, work engagement.

Hypothesis 2 proposed that job crafting would mediate the relationship between empowering leadership and work engagement. As reported in Table 3, the indirect effect of empowering leadership on work engagement through job crafting was significant (indirect effect = 0.184, *p* < 0.001, 95% CI = [0.116, 0.263], *β* = 0.182). Hypothesis 2 was therefore supported.

Hypothesis 3 proposed that role breadth self-efficacy would moderate the relationship between empowering leadership and job crafting. The interaction between empowering leadership and role breadth self-efficacy significantly predicted job crafting (B = 0.346, *p* < 0.001, *β* = 0.271). As illustrated in Figure 2, the positive relationship between empowering leadership and job crafting was stronger at high levels of role breadth self-efficacy (B = 0.619, *t* = 11.829, *p* < 0.001, *β* = 0.613) than at low levels of role breadth self-efficacy (B = 0.073, *t* = 1.751, *p* = 0.081, *β* = 0.072). Thus, Hypothesis 3 was supported.

**Figure 2:**
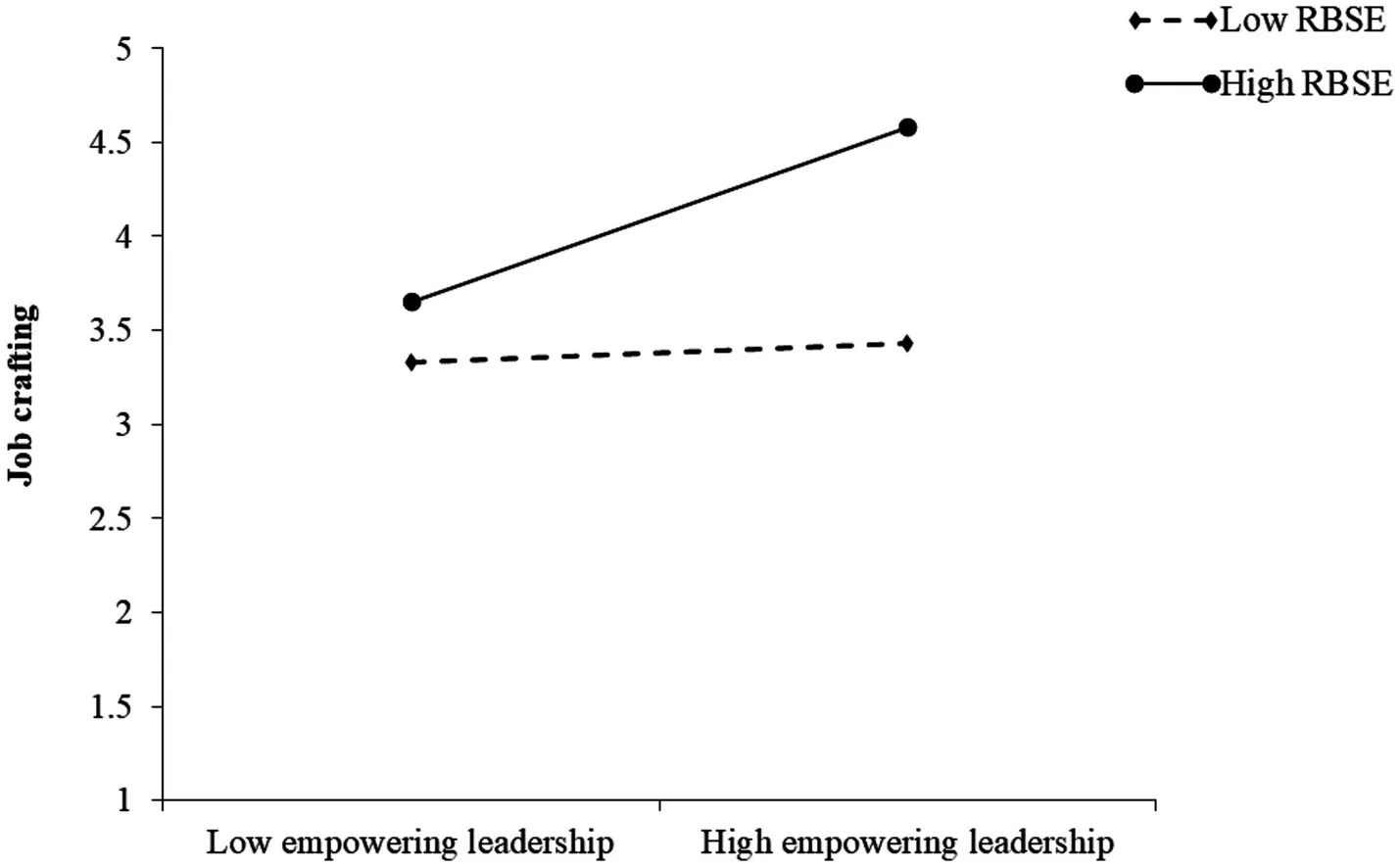
The interactive effect of empowering leadership and role breadth self-efficacy on job crafting.

Hypothesis 4 predicted that role breadth self-efficacy would condition the indirect relationship between empowering leadership and work engagement through job crafting. As shown in Table 3, the conditional indirect effect was positive and significant when role breadth self-efficacy was high (B = 0.367, SE = 0.047, 95% CI = [0.275, 0.461], *β* = 0.363), but weaker and not statistically significant when role breadth self-efficacy was low (B = 0.043, SE = 0.035, 95% CI = [−0.015, 0.121], *β* = 0.043). Consistent with Hayes (2015), the index of moderated mediation was significant (index = 0.205, 95% CI = [0.134, 0.262]), supporting Hypothesis 4. This contrast indicates that the indirect association between empowering leadership and work engagement through job crafting is substantively stronger when RBSE is high, underscoring the practical importance of teachers’ confidence in undertaking broader roles.

As a robustness check, we re-estimated the hypothesized model without gender, age, and organizational tenure as control variables. The focal estimates were virtually unchanged. For example, the direct association between empowering leadership and work engagement changed only from B = 0.199 to B = 0.196, the interaction effect changed from B = 0.346 to B = 0.348, and the index of moderated mediation changed from 0.205 to 0.206. These results indicate that the inclusion of the demographic controls did not materially affect the findings.

## 5 Discussion

Drawing on JD-R theory, this study examined how empowering leadership is associated with university teachers’ work engagement through contextual job resources, job crafting, and RBSE. The findings support a conditional resource pathway: empowering leadership was related to higher work engagement, job crafting explained part of this relationship, and the indirect relationship through job crafting was stronger at higher levels of RBSE. The central implication is that university teachers’ engagement should not be treated only as a direct response to leader encouragement or as a stable individual attitude. It is better understood as a resource-based work-design process in which contextual resources, personal efficacy, and proactive behavior operate together.

This interpretation is important because academic work combines professional autonomy with strong institutional pressure. Research on higher education shows that faculty well-being is shaped by workload, performance demands, autonomy, support, and resource availability (Mudrak et al., 2018; Naidoo-Chetty and du Plessis, 2021). Our findings suggest that empowering leadership is positively associated with teachers’ job crafting. This pattern is consistent with a theoretical account linking leader-provided participation, confidence, and autonomy to teachers’ proactive work redesign. Following the JD-R logic that job resources energize engagement by supporting goal attainment and development (Bakker and Demerouti, 2017), empowering leadership can be interpreted as a social and organizational resource in universities. This complements meta-analytic evidence that resources are central to engagement (Mazzetti et al., 2023) and shows more specifically how leadership resources may operate in academic work.

The mediating role of job crafting provides a potential behavioral explanation for this relationship. Empowering leadership was associated with teachers’ efforts to redesign tasks, relationships, and work meanings, which in turn were linked to engagement. This aligns with Kim and Beehr (2020), who showed that job crafting can transmit the effects of empowering leadership to employee outcomes, but the academic context gives the mechanism a distinctive interpretation. University teachers can craft their work by redesigning classroom interaction, seeking research collaborators, strengthening student mentoring, or reframing service as contribution to the academic community. Zhang and Parker’s (2019) integrative review emphasizes that job crafting includes multiple proactive strategies rather than one generic behavior, and recent research among university professors shows that approach-oriented crafting can be associated with prosocial service behavior (Ortega-Egea et al., 2025). Teacher-focused JD-R research further suggests that different crafting behaviors relate to teacher self-efficacy and engagement in nuanced ways (Huang et al., 2025). The present findings indicate that greater job crafting is associated with higher work engagement.

The moderating role of RBSE adds an important boundary condition. Empowerment is not self-executing: it provides room for action, but teachers still need confidence that they can act beyond narrowly defined role expectations. Parker (1998) introduced RBSE to capture this broader efficacy belief, and JD-R research positions self-efficacy-related personal resources as factors that shape how employees perceive, mobilize, and benefit from available job resources (Xanthopoulou et al., 2007). In the present model, the positive relationship between empowering leadership and job crafting was stronger among teachers with higher RBSE. This interpretation is compatible with evidence from Hao et al. (2018) that RBSE shapes when empowering leadership produces stronger performance outcomes, and with Rodrigues and Rebelo’s (2023) evidence linking RBSE and innovative work behavior to promotion prospects. In universities, RBSE may determine whether an academic leader’s invitation to participate is experienced as a professional opening or as an uncertain extra demand. This interpretation is also consistent with recent evidence that followers with low RBSE may experience empowering leadership as hindrance-related stress rather than as an enabling resource (Sun et al., 2025).

### 5.1 Theoretical implications

First, this study advances JD-R theory in higher education by specifying a conditional resource pathway rather than a simple resource-engagement link. Bakker and Demerouti (2017) define job resources as aspects of work that support goal attainment, reduce demands, and promote development, and longitudinal and meta-analytic JD-R studies show that resources reliably predict engagement (Lesener et al., 2019; Mazzetti et al., 2023). Recent JD-R theorizing further emphasizes that employees are not only exposed to job demands and resources but also actively shape them through proactive behaviors such as job crafting, and that personal resources influence how employees mobilize available resources (Bakker and Demerouti, 2024; Bakker et al., 2023). The present findings extend this logic to university teachers by showing that empowering leadership can operate as a contextual job resource in academic settings. This is theoretically meaningful because universities are not conventional hierarchical workplaces: teachers often possess professional autonomy, yet they also face institutional constraints, workload intensification, and multiple role expectations. Evidence on faculty well-being and academic staff engagement in JD-R frameworks has emphasized these mixed demands and resources across individual, team, and organizational levels (Cao and Zhang, 2022; Mudrak et al., 2018; Naidoo-Chetty and du Plessis, 2021; Whitsed et al., 2025). The present study therefore refines JD-R theory by showing that leadership resources matter even where autonomy is formally present, as reflected in the observed associations among empowering leadership, job crafting, and engagement.

Second, this study extends empowering leadership research by clarifying how followers’ RBSE conditions the job crafting pathway to work engagement. Kim and Beehr (2020) modeled empowering leadership and core self-evaluations as parallel antecedents of job crafting in explaining employee well-being. Our findings extend this account by showing that both the leadership–crafting association and the indirect association with engagement were stronger at higher levels of RBSE. Personal resources thus appear relevant not only as antecedents of proactive work redesign but also as conditions shaping employees’ responsiveness to leadership resources. Thun and Bakker (2018) found that optimism strengthened the associations of empowering leadership with increasing structural resources and challenging demands. We build on this contingency account by identifying role-specific efficacy as a personal resource relevant to leadership-related crafting. Whereas optimism concerns generalized expectations of favorable outcomes, RBSE concerns perceived capability to undertake broader work activities. This distinction gives the contingency argument greater behavioral specificity: the opportunities for initiative afforded by empowering leadership may be more readily acted upon when followers feel capable of assuming broader responsibilities. Finally, Mao et al. (2026) positioned RBSE as a mediator between empowering leadership and job crafting, emphasizing efficacy beliefs as a psychological mechanism linking leadership and proactive work redesign. Our findings complement this account by identifying RBSE as a boundary condition of the leadership–crafting relationship. The theoretical extension concerns the function of personal resources: beyond being resources that leadership may foster, they may shape how employees respond to empowerment. Taken together, these comparisons refine JD-R-based explanations of empowering leadership by specifying how confidence in broader roles complements leadership-provided opportunities within the job crafting pathway to engagement.

Third, this study enriches job crafting theory by locating crafting within professional academic work. Job crafting research increasingly treats crafting as differentiated, contextual, and motive-laden rather than as a single adjustment behavior. Zhang and Parker (2019) distinguish multiple forms and levels of crafting, while Tims et al. (2022) call for closer attention to context, social relationships, and longitudinal processes. Demerouti’s (2026) recent review further emphasizes job crafting’s positive and negative outcomes, contextual mechanisms, and the need for balanced crafting to avoid additional workload and burnout. University teachers provide a useful context because their tasks are broad, interdependent, and meaning-laden. The present findings show that job crafting is not simply an individual preference for modifying work. It is a context-sensitive behavior that is associated with empowering leadership. This view is consistent with Lazazzara et al.’s (2020) qualitative synthesis, which portrays job crafting as a process of reinventing work through interaction between individuals and their work environments. By showing that job crafting mediates the relationship between empowering leadership and engagement, the study highlights the associations among contextual resources, proactive work redesign, and engagement in academic work.

Fourth, the study advances research on personal resources by positioning RBSE as a first-stage condition in a moderated mediation model. Following Parker’s (1998) original formulation, RBSE captures confidence in carrying out broader and more proactive roles. Within JD-R theory, personal resources such as self-efficacy are important because they shape how employees perceive their work environment, mobilize job resources, and sustain motivational outcomes (Xanthopoulou et al., 2007). In this study, RBSE moderates the relationship between empowering leadership and job crafting, as well as the indirect relationship with work engagement. This complements evidence that RBSE conditions the effects of empowering leadership on work outcomes (Hao et al., 2018). The implication for JD-R theory is that personal resources may help explain why contextual job resources differ in their associations with job crafting and engagement. In academic work, the indirect relationship between empowering leadership and engagement through job crafting is stronger among teachers who feel capable of acting beyond narrow role boundaries.

### 5.2 Practical implications

First, universities should treat empowering leadership as a concrete leadership capability rather than as a general slogan. Department heads, deans, program directors, and research-team leaders need to know how to invite meaningful participation, communicate confidence, explain the purpose of work, and remove unnecessary constraints. These are the core behaviors in Ahearne et al.’s (2005) empowering leadership framework, and JD-R-based leadership research similarly suggests that leaders can enhance engagement by creating autonomy-supportive, competence-building, socially connected, and meaningful work conditions (Schaufeli, 2021; Tuckey et al., 2012). For universities, leadership development should therefore move beyond administrative compliance training. Academic leaders should be trained to design participatory decision processes, give confidence-building and developmental feedback, protect space for pedagogical and research initiative, and distinguish genuine empowerment from the downward transfer of responsibility.

Second, universities should support job crafting as part of academic professional development. Teachers often know where their work could be redesigned, but they may lack time, legitimacy, or peer support to act on that knowledge. Job crafting research shows that employees craft work through task, relational, and cognitive changes (Zhang and Parker, 2019), and Tims et al. (2022) emphasize that crafting should be understood in relation to social context and work conditions. Intervention research suggests that job crafting can be developed through structured reflection, goal setting, and experimentation (Oprea et al., 2019), while recent review evidence cautions that crafting should remain balanced so that it does not intensify workload or burnout (Demerouti, 2026). Practical support could include peer coaching groups, course-redesign clinics, mentoring circles, and guided reflection sessions in which teachers identify one manageable change in task design, collaboration, feedback seeking, or work meaning. Such initiatives should be tied to real workload and resource arrangements, because crafting is unlikely to flourish when teachers are asked to be proactive while their basic job demands remain unmanageable.

Third, universities should cultivate RBSE deliberately. Given that the positive relationship between empowering leadership and job crafting was stronger at higher levels of RBSE, institutions should create graduated opportunities for teachers to act beyond narrow role boundaries. Parker’s (1998) work suggests that job enrichment and organizational interventions can strengthen RBSE, while research links RBSE to proactive behavior, innovative work behavior, and career-relevant outcomes (Rodrigues and Rebelo, 2023; Strauss et al., 2009). Universities can build this resource by giving teachers scaffolded opportunities to lead curriculum improvement, participate in interdisciplinary projects, mentor junior colleagues, coordinate student-support initiatives, or contribute to departmental problem solving. The practical point is not to push every teacher into broader roles at once, but to provide supported mastery experiences, feedback, and visible examples of successful role expansion that make broader role behavior feel attainable.

Fourth, universities should pair empowerment with clarity, resources, and workload protection. Empowering leadership can become counterproductive when it is experienced as vague delegation or as an invitation to absorb additional responsibilities without support. Double-edged accounts of empowering leadership suggest that empowerment can be enabling as well as burdening, particularly when added discretion increases responsibility without adequate capacity, clarity, or support (Cheong et al., 2016; Rai et al., 2024; Sun et al., 2025). Higher education research likewise shows that workload and resource conditions shape academic engagement (Naidoo-Chetty and du Plessis, 2021; Whitsed et al., 2025). Academic leaders should therefore clarify decision boundaries, provide time and resources for innovation, monitor workload distribution, and offer more scaffolding for teachers with lower RBSE. Empowerment is most useful when teachers know where they have discretion, what support is available, and how their crafted initiatives connect to institutional goals.

## 6 Limitations and future research

Several limitations should be acknowledged. First, the study relies on cross-sectional, self-report data, which limits causal inference and raises the possibility of common method variance. Although the proposed model is theoretically grounded, cross-sectional tests of mediation cannot establish temporal precedence and may bias estimates of indirect effects (Maxwell et al., 2011), and same-source survey designs may be affected by method-related variance (Podsakoff et al., 2012). Future longitudinal studies should repeatedly assess empowering leadership, job crafting, RBSE, and work engagement across at least three waves to examine temporal ordering and reciprocal relationships, accounting for prior levels and incorporating multiple data sources where appropriate. Time-lagged diary studies could examine whether within-person fluctuations in empowering leadership precede changes in job crafting and engagement, and whether these associations vary with RBSE. Randomized interventions targeting empowering leadership, with RBSE assessed beforehand, could strengthen causal inference regarding leadership effects on subsequent job crafting and engagement. Building on multilevel leadership research (Tuckey et al., 2012), future studies could sample multiple teachers under each department head to distinguish individual perceptions from department-level empowering leadership, where aggregation is justified. Such studies could test whether individual RBSE moderates the association between departmental empowering leadership and teachers’ job crafting, thereby conditioning the indirect association with engagement.

Second, the use of convenience sampling may have introduced selection bias, as teachers who were more accessible or willing to participate may differ from those who were not included. Therefore, caution is needed when generalizing the findings to the broader population of Chinese university teachers. The study focuses on university teachers, and the meaning of empowering leadership may vary across institutional missions, disciplines, career stages, and national higher education systems. Although this context is theoretically appropriate because academic work combines autonomy, discretion, and institutional pressure, it also limits the generalizability of the findings. Future research could compare research-intensive universities, teaching-oriented institutions, vocational colleges, and private universities to examine whether the same resource pathway holds under different governance and workload conditions. It would also be useful to test whether the model differs across disciplines, academic ranks, tenure status, and national cultures, because teachers’ discretion and exposure to performance demands may vary substantially across these groups.

Third, the model centers on job crafting and RBSE, but other mechanisms and boundary conditions may also matter. Future studies could distinguish task, relational, cognitive, promotion-focused, and prevention-focused crafting to examine whether different forms of crafting have different implications for engagement. This distinction is important because resource- and challenge-oriented crafting may be more engagement-enhancing than avoidance-oriented crafting. Future research could also examine moderators such as psychological safety, proactive personality, workload, and departmental climate, and extend the outcome side to teaching quality, research collaboration, organizational citizenship, retention, and student-related outcomes. Such extensions would help clarify whether empowering leadership and job crafting not only improve teachers’ motivational states but also contribute to broader educational and institutional outcomes.

## Data Availability

The raw data supporting the conclusions of this article will be made available by the authors, without undue reservation.
